# The Prognostic Significance of Tumor-Infiltrating Lymphocytes, PD-L1, BRCA Mutation Status and Tumor Mutational Burden in Early-Stage High-Grade Serous Ovarian Carcinoma—A Study by the Spanish Group for Ovarian Cancer Research (GEICO)

**DOI:** 10.3390/ijms241311183

**Published:** 2023-07-06

**Authors:** David Pizarro, Ignacio Romero, Belén Pérez-Mies, Andrés Redondo, Tamara Caniego-Casas, Irene Carretero-Barrio, Eva Cristóbal, Ana Gutiérrez-Pecharromán, Ana Santaballa, Emanuela D’Angelo, David Hardisson, Begoña Vieites, Xavier Matías-Guiu, Purificación Estévez, Eva Guerra, Jaime Prat, Andrés Poveda, José Antonio López-Guerrero, José Palacios

**Affiliations:** 1 Pathology Department, University Hospital Ramón y Cajal, IRYCIS, 28034 Madrid, Spain; dapizaroo87@gmail.com (D.P.); bperezm@salud.madrid.org (B.P.-M.); tamara.caniego@salud.madrid.org (T.C.-C.); irene.carretero@salud.madrid.org (I.C.-B.); 2 Instituto Valenciano de Oncología, 46009 Valencia, Spain; iromero@fivo.org; 3 Spanish Group for Investigation on Ovarian Cancer (GEICO), 28003 Madrid, Spain; aredondo12@gmail.com (A.R.); santaballa_ana@gva.es (A.S.); puriestevez@gmail.com (P.E.); evamaria.guerra@salud.madrid.org (E.G.); apoveda@initiaoncologia.com (A.P.); 4 Biomedical Research Network in Oncology (CIBERONC), Instituto de Salud Carlos III, 28029 Madrid, Spain; evamaria.cristobal@salud.madrid.org (E.C.); david.hardisson@salud.madrid.org (D.H.); xmatias@bellvitgehospital.cat (X.M.-G.); 5 Faculty of Medicine, University of Alcalá, 28801 Alcalá de Henares, Spain; 6 Oncology Department, University Hospital La Paz, IdiPAZ, 28046 Madrid, Spain; 7 Instituto de Investigación Sanitaria del Hospital Universitario La Paz, 28029 Madrid, Spain; 8 Faculty of Medicine, Autonomous University of Madrid, 28029 Madrid, Spain; 9 Pathology Department, University Hospital Getafe, 28905 Getafe, Spain; agpecharroman@salud.madrid.org; 10 Oncology Department, University Hospital La Fe, 46026 Valencia, Spain; 11 Department of Medical, Oral, and Biotechnological Sciences, University “G.D’Annunzio” of Chieti-Pescara, 66013 Chieti, Italy; emanuela.dangelo@unich.it; 12 Pathology Department, University Hospital La Paz, 28046 Madrid, Spain; 13 Pathology Department, University Hospital Virgen del Rocío, 41013 Sevilla, Spain; mb.vieites.sspa@juntadeandalucia.es; 14 Pathology and Medical Oncology Departments, Hospital Universitari Arnau de Vilanova, IRBLLEIDA, University of Lleida, 25003 Lleida, Spain; 15 Pathology Department, Hospital Universitari de Bellvitge, IDIBELL, University of Barcelona, 08007 Barcelona, Spain; 16 Oncology Department, University Hospital Virgen del Rocío, 41013 Sevilla, Spain; 17 Seville Biomedical Research Institute (IBIS), 41013 Sevilla, Spain; 18 Oncology Department, University Hospital Ramón y Cajal, IRYCIS, 28034 Madrid, Spain; 19 Pathology Department, Emeritus Faculty, Autonomous University of Barcelona, 08193 Barcelona, Spain; jpratdl@gmail.com; 20 Initia Oncología, Hospital Quironsalud Valencia, 46010 Valencia, Spain

**Keywords:** high-grade serous ovarian carcinoma, early stage, PD-L1, *BRCA*, TMB

## Abstract

Early stages are under-represented in studies on the molecular and immune features of high-grade serous ovarian carcinoma (HGSOC), and specific studies focused on early-stage HGSOC are required for a better prognostic stratification and to personalize chemotherapy. The aim of this study was to determine the prognostic significance of CD8+ and CD4+ tumor-infiltrating lymphocytes (TILs), tumoral cell PD-L1 expression, *BRCA* mutational status and tumor mutation burden (TMB) in early-stage HGSOC. A retrospective study was performed on stage I and II HGSOC from the Molecular Reclassification of Early Stages of Ovarian Cancer (RECLAMO) cohort from the Spanish Group of Ovarian Cancer Research (GEICO). Centralized histological typing was performed based on morphological and immunohistochemical features. Intraepithelial (i) and stromal (s) CD8+ and CD4+ T cells and PD-L1 were evaluated on tissue microarrays by immunohistochemistry. *BRCA1* and *BRCA2* mutation status and TMB were analyzed in tumor DNA using next-generation sequencing. The study included 124 tumors. High iCD8+ (>20 TILs/core), low/intermediate CD4+ (<20 TILs/core) and high CD8+/CD4+ ratio (>35/core) were associated with favorable outcomes. Tumor cell PD-L1 expression (TPS ≥ 1) was present in only 8% of tumors. In total, 11 (16%) and 6 (9%) out of 69 HGSOC tested carried pathogenic or likely pathogenic *BRCA1* or *BRCA2* mutations, respectively. Median TMB of 40 tumors analyzed was 5.04 mutations/Mb and only 6 tumors had 10 or more mutations/Mb. *BRCA* status and TMB were not associated with TILs or prognosis. When compared with studies on advanced HGSOC, our results suggested that prognostic variables differed according to stage and that more studies focused on early stages of HGSOC are needed to better stratify these tumors.

## 1. Introduction

There are five main histological types of ovarian carcinoma (OC): high-grade serous ovarian carcinoma (HGSOC), low-grade serous ovarian carcinoma (LGSOC), endometrioid ovarian carcinoma (EOC), clear cell ovarian carcinoma (CCOC) and mucinous ovarian carcinoma (MOC). These histological types differ in their precursor lesions, morphology, underlying molecular alterations, prognosis and response to treatment [1,2,3,4]. HGSOC is the most frequent and aggressive histological type of OC. Although most cases present in advanced stages, about 10–15% of patients debut in early stages (stage I and II) [5]. Due to their low frequency, there are few studies that analyze the molecular features and prognostic variables of early stage HGSOC [6].

Patients with HGSOC, even in early stages, are considered to have a high risk of relapse, so adjuvant chemotherapy is generally recommended. A better evaluation of this risk would allow chemotherapy to be personalized and reduce costs and treatment side effects in patients with low risk of relapse. For this reason, prognostic markers are needed to stratify patients and select those who will benefit from chemotherapy [7]. 

Some biomarkers related to the immune microenvironment or immune response have been analyzed as prognostic markers in OC. Nearly 20 years ago, Zhang et al. [8] reported for the first time an association between an increased number of intraepithelial (i) CD3+ cells and good prognosis in HGSOC. Since then, several studies have reported that prognosis was more directly related with the number of iCD8+ T cells [9,10]. However, most of these studies were focused on advanced HGSOC, and information regarding early stages has been very limited [11]. 

PD-L1 is currently used as a response predictor to immune checkpoint inhibitors (ICIs) in several types of cancers. Moreover, some studies have also tested its role as a possible prognostic marker in OC [12]. However, most studies were based on the analysis of advanced-stage tumors and have reported discordant results regarding not only the frequency of PD-L1 expression in tumor cells, but also whether or not PD-L1 is a marker of prognosis.

*BRCA1* mutations have been related with increased immune cell infiltration in HGSOC [11,13]. In addition, advanced tumors with *BRCA* mutations seem to have a more favorable prognosis [14,15,16], probably due to the fact that they respond better to chemotherapy and PARP inhibitors (PARPi) due to homologous repair deficiency (HRD). The role of tumor mutational burden (TMB) as a prognostic or predictive marker has been scarcely studied in HGSOC [17,18,19,20] and its exact role as a possible biomarker in early-stage HGSOC remains to be established. Again, most studies reporting *BRCA* status and TMB as biomarkers were enriched in advanced-stage tumors.

A major limitation of most studies analyzing immune biomarkers in OC is that the histological type was assigned using only morphological criteria. Thus, after a centralized review using immunohistochemistry to complement morphological evaluation, it has been estimated that 20% of OC are assigned to a different histological type [2,21]. In this study, CD4+ and CD8+ TILs, PD-L1 expression, *BRCA* status and TMB were analyzed in a large series of early-stage HGSCs, in which histotyping had been performed using both morphological and immunohistochemical criteria, to analyze their impact on prognosis.

## 2. Results

### 2.1. Clinicopathological and Immunohistochemical Features

The series included 124 HGSOC, with a diagnosis based on both morphological and immunohistochemical criteria, as previously reported [2]. The diagnostic algorithm, which takes into account the expression of WT1, p53, Napsin A and PR was used to assist the classification of samples [21]. HGSOC were characterized by WT1+ and p53 abnormal pattern. In addition, most HGSC (87, 70.16%) showed diffuse nucleocytoplasmic expression of p16.

Due to the retrospective nature of the study and the multiple centers involved, some important clinical variables, such as family history, comorbidities, intraoperative findings or ascites cytology, were missing in our database. However, most patients were surgically staged and received a similar chemotherapy treatment. The main characteristics of this series are presented in Table 1 and Supplementary Table S1. HGSOC was diagnosed with a mean age of 57 years. Nearly half of the patients (59, 47.6%) were stage IC at diagnosis. Most patients (110, 92.4%) received platinum-based chemotherapy.

We evaluated the presence of CD4+ and CD8+ lymphocytes in the epithelium (iCD4+ and iCD8+) and stromal compartments (sCD4+ and sCD8+), counting the number of positive cells in each compartment. Each type of TIL was divided into the following three categories: no TILs, low/moderate TILs (1 to 19 lymphocytes) and high TILs (>20). The CD8+/CD4+ ratio was calculated in each compartment. 

**Figure 1:**
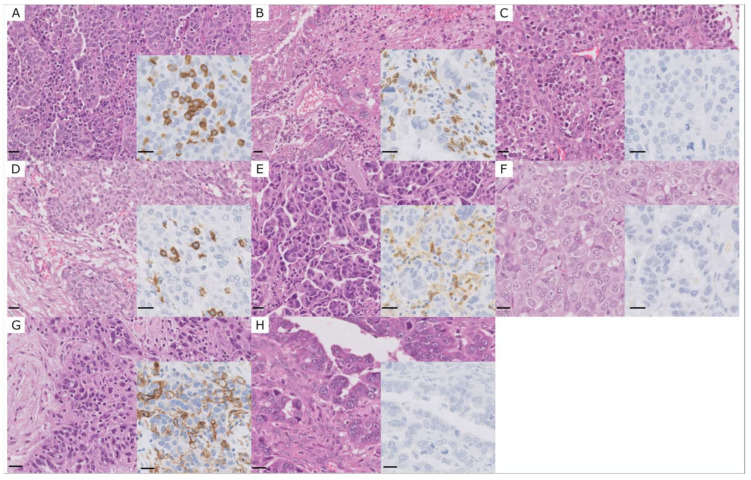
(**A**) Case with high iTILs CD8+. Inset: CD8 immunohistochemistry. (**B**) Case with high sTILs CD8+. Inset: CD8 immunohistochemistry. (**C**) Case with no CD8+ lymphocytes. Inset: CD8 immunohistochemistry. (**D**) Case with high iTILs CD4+. Inset: CD4 immunohistochemistry. (**E**) Case with high sTILs CD4+. Inset: CD4 immunohistochemistry. (**F**) Case with no CD4+ lymphocytes. Inset: CD4 immunohistochemistry. (**G**) PD-L1-positive case. Inset: PD-L1 immunohistochemistry. (**H**) PD-L1-negative case. Inset: PD-L1 immunohistochemistry. Scale bar 20 µm.

The most abundant lymphocyte population per core was iTILs-CD8+ (mean 49.55), followed by sTILs-CD8+ (mean 31.43), sTILs-CD4+ (mean 18) and iTILs-CD4+ (5.95) (Table 2 and Figure 1). The proportion of tumors with negative, low/moderate and high TILs is presented in Table 2. Whereas 60.8% of HGSOC in this series had high iCD8+TILs, only 10,7% of tumors had high iCD4+. The mean iCD8+/iCD4+ ratio was 35.75 and the median iCD8+/iCD4+ ratio was 15.50. No differences in any type of TILs or TIL ratios were observed according to stage (Supplementary Figure S1) or age.

**Figure 2:**
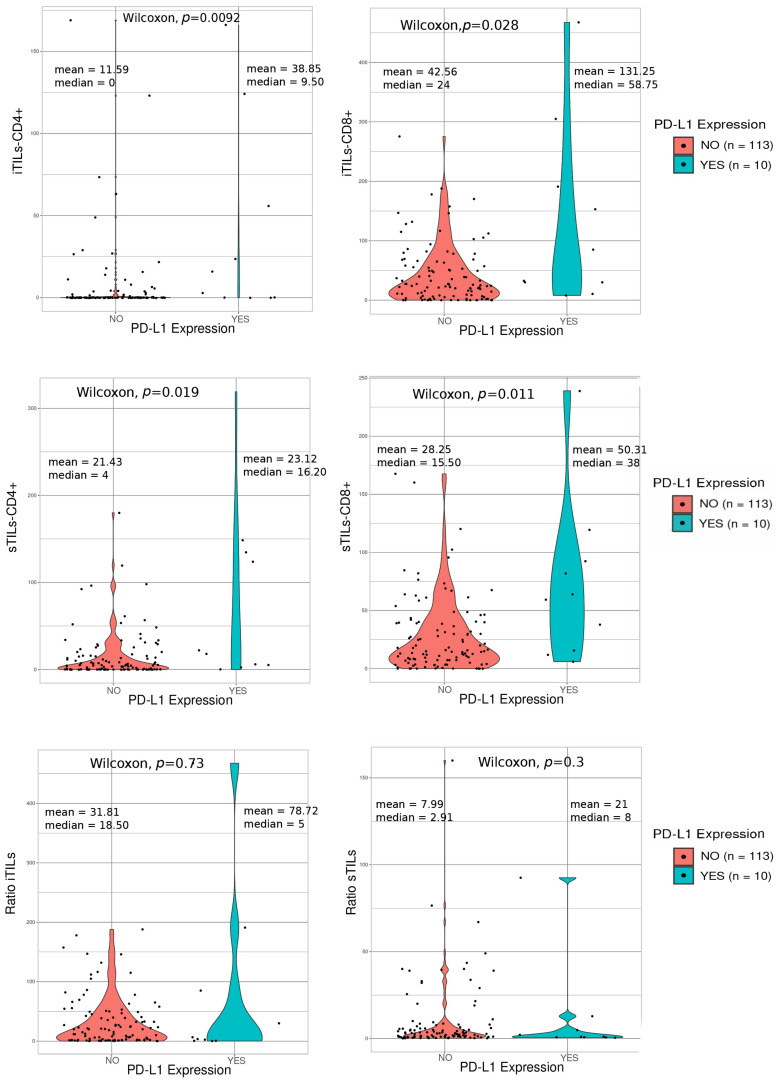
Wilcoxon tests showing the association between TILs and PD-L1 expression. No (negative PD-L1), Yes (positive PD-L1).

Only 10 of 123 assessed tumors (8.1%) expressed PD-L1 in more than 1% of cells (TPS ≥ 1) and were considered positive. No association between PD-L1 expression and age or stage was observed. In contrast, PD-L1 expression was associated with TILs, and the number of all TIL categories was significantly higher in PD-L1-positive tumors (Figure 2).

### 2.2. BRCA1 and BRCA2 Mutational Status

DNA was extracted from all tumors, but only 72 rendered DNA of enough quality for molecular studies, and 69 of them gave valid results for *BRCA* analysis. In total, 11 (16%) and 6 (9%) out of 69 HGSOC carried pathogenic or likely pathogenic *BRCA1* or *BRCA2* mutations, respectively (Table 1 and Supplementary Table S2). No statistically significant associations were found between *BRCA* status and age or stage. Mutations in any or both *BRCA* genes were not associated with the number of TILs (Supplementary Figures S2–S4) or PD-L1. 

### 2.3. Tumor Mutational Burden (TMB)

TMB analysis was performed in 62 tumors in which DNA was available after *BRCA* analysis. However, due to DNA quality, valid results were only obtained in 40 tumors (Table 1 and Supplementary Table S3). The median number of mutations/Mb was 5.04. Six tumors (15%) had 10 or more than 10 mutations/Mb. Two different thresholds were used to define high TMB, the median number of mutations/MB and >10 mutations/Mb. No association was found between TMB and any clinical variable, TILs (Supplementary Figure S5), PD-L1 or *BRCA* status (Supplementary Figure S6) using either of the two defined thresholds. 

### 2.4. Survival Analysis

**Figure 3:**
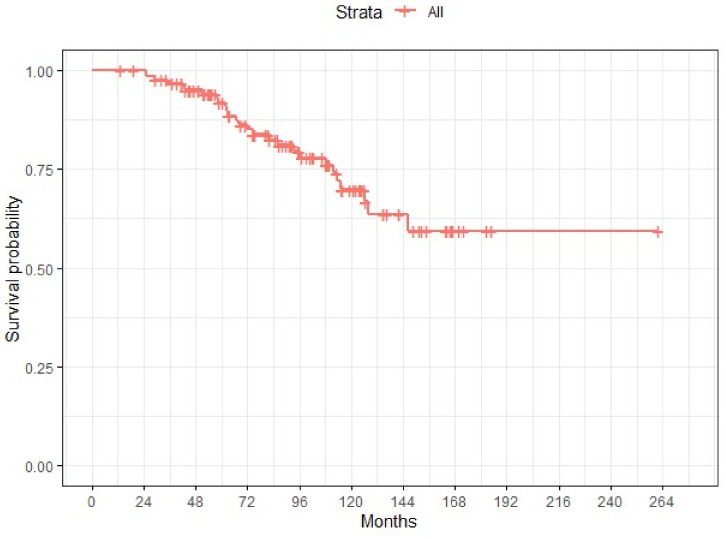
Kaplan–Meier estimate of overall survival for the whole cohort.

**Figure 4:**
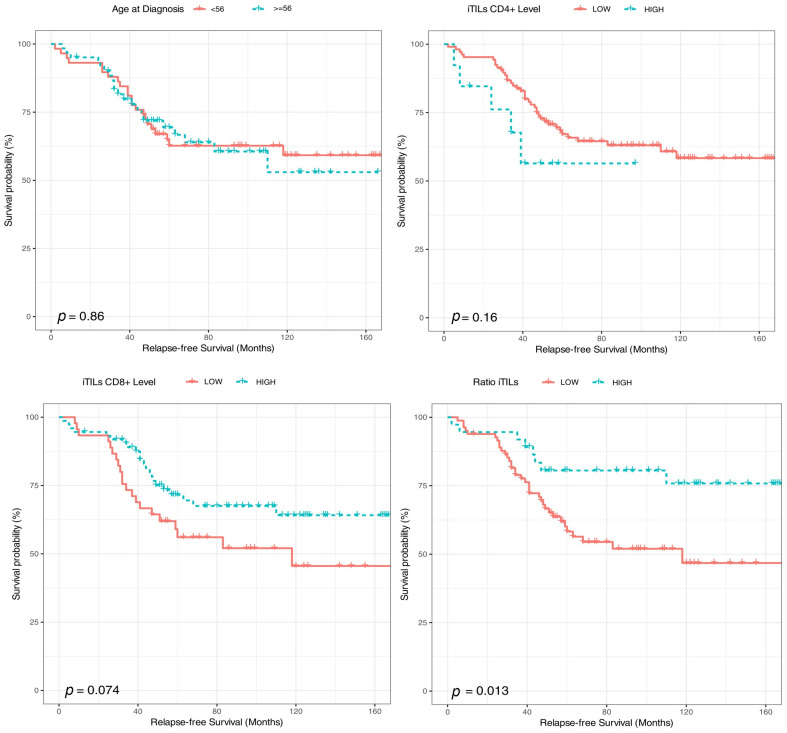
Kaplan–Meier estimates of relapse-free survival according to age at diagnosis, iTILs CD4+, iTILs CD8+ and iTILs ratio.

**Figure 5:**
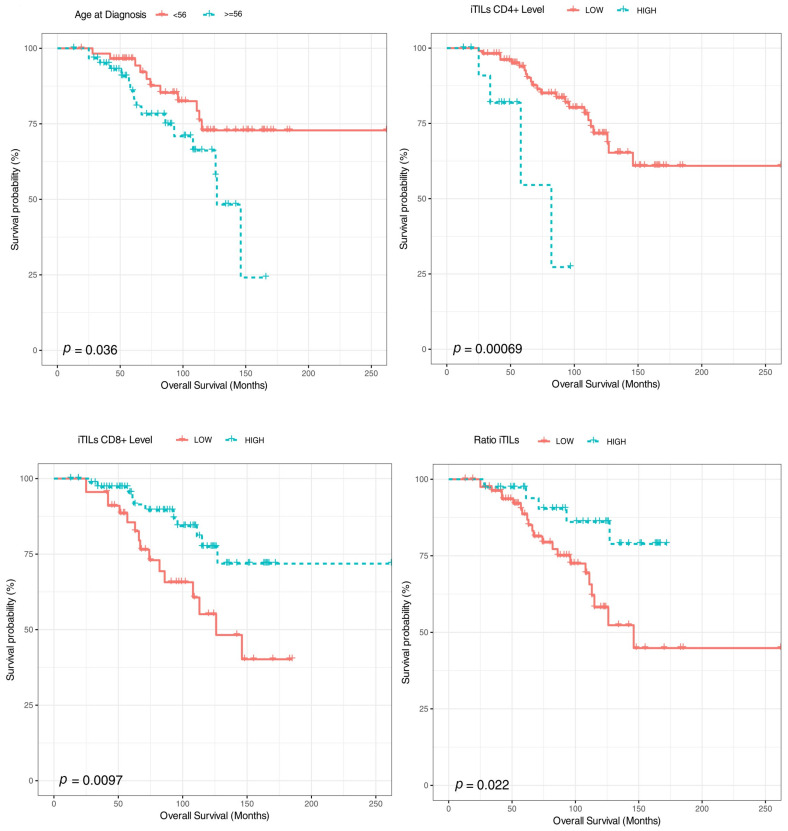
Kaplan–Meier estimates of overall survival, according to age at diagnosis, iTILs CD4+, iTILs CD8+ and iTILs ratio.

**Figure 6:**
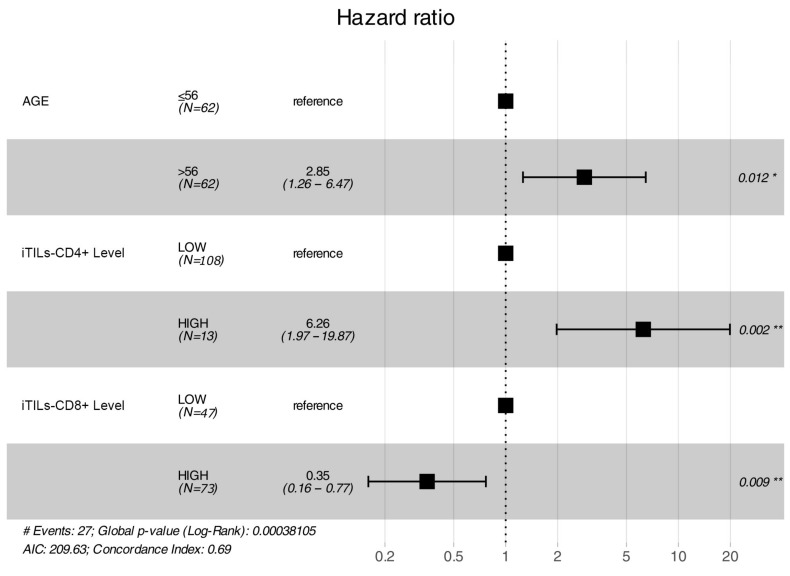
Multivariate Cox model showing age, iTILs CD4+ (LITCD4_Level) and iTILs CD8+ (LITCD8_Level) as independent prognostic factors for overall survival. The asterisk indicates statistical significance: (*) *p*-value = 0.01 to 0.05 and (**) *p*-value < 0.01.

After a median follow-up of 85 months, 44 (35.5%) patients relapsed and 27 (21.7%) died (Figure 3). Age at diagnosis, iCD8+ (as a categorical variable), iCD4+ (as a categorical variable) and the ratio iCD8+/iCD4+ were associated with overall survival in the univariate analysis. Thus, patients younger than 56 years and tumors with high iTILs-CD8+ (>20), low/moderate iTILS-CD4+ (<20) and higher iCD8+/iCD4+ ratios (>35) had a longer survival (Figure 4 and Figure 5). In the multivariate analysis, only age, iCD8+ and iCD4+ remained as independent prognostic factors for overall survival (Figure 6). None of the other variables analyzed (stage, PD-L1, TMB, *BRCA1* and *BRCA2* mutations and *BRCA* status) were significantly associated with relapse-free survival or overall survival (Supplementary Figures S7–S10). 

## 3. Discussion

In this series of early-stage HGSOC, centralized histotyping was performed using both histological and immunohistochemical features. We observed a prognosis similar to that reported in the literature for early-stage HGSOC, with an overall survival at 120 months of around 70% [22,23]. Some studies report slightly worse outcomes, with around 50% survival at 120 months [24].

We observed that high iCD8+ infiltration was associated to a favorable prognosis. Our results confirmed the observation of the only previously reported study with a large number of early-stage HGSOC, as well as the results of most previous series in advanced-stage HGSOC [9,10,11]. From a clinical and diagnostic point of view, the best TIL cut-off to stratify prognosis remains to be established, since some studies, such as the one presented here, have used TMA, whereas others have used whole tissue sections. In addition, the cut-off was variable among studies. To take into account intratumoral heterogeneity in TILs across cores per patient, the average value of the two core counts was used, as reported by other authors [25]. In contrast, Goode et al. [11] used the maximum TIL count per high-power microscopic field. At present, whether evaluating hot-spot areas of TILs provides better prognostic information than the average count has not been established. In other tumors, such as breast cancer, where TILs have been associated with prognosis and therapy response, it is recommended to evaluate average TILs. As previously suggested by Goode et al. [11], a practical and robust scoring system should be developed for TILs in HGSOC.

In addition to increased iCD8+, we observed that the prognosis was more favorable in tumors with low/moderate iCD4+ infiltration. The role of iCD4+ in early-stage HGSOC has not yet been established. The study of Goode et al. [11] did not include CD4 analysis and other studies mainly focused on advanced stages. Whereas Hao et al. [9] reported in their meta-analysis that increased iCD4 was associated with favorable prognosis, this association was not observed in the meta-analysis by Li et al. [10]. The unfavorable effect of CD4+ cells on prognosis in advanced HGSOC reported in some series has been related with an increase in FOXP3+ cells, although not all studies have confirmed this association [10]. Preston et al. [26] and Sato et al. [27] suggested that the effector/suppressor ratio CD8+/CD4+ may be a more important indicator of outcome than individual cell count. Accordingly, we found that high CD8+/CD4+ ratio was associated with a favorable prognosis in the univariate analysis, but this association was lost in the multivariate analysis.

We observed that only 8% of early-stage HGSOC in this series expressed PD-L1 in tumor cells and that this expression was not associated with prognosis. Series that analyzed PD-L1 in HGSOC are heterogeneous in terms of clinicopathological features, staining assays, cut-off values and the evaluation of tumor cells, inflammatory cells or both [28,29,30,31,32,33,34]. In previous studies, the percentage of positive tumor cells ranged from 24% [28] to 88% [29]. The low percentage of tumor cell positivity in our series is probably related to the analysis of only early-stage tumors. Thus, Wang et al. [28] reported that 24% of HGSC (n = 107) in their series expressed PD-L1 in tumor cells, but this percentage dropped to 7.7% in early-stage tumors (n = 26). In addition, a recent meta-analysis by Zhang and Yang reported that PD-L1 tumor cell positivity was correlated to advanced FIGO stages [30]. 

The prognostic significance of PD-L1 in ovarian cancer is controversial, with studies indicating its association with both a favorable and unfavorable prognosis. Some series [12] and the meta-analysis by Zhang and Yang [30] that included all stages suggested that PD-L1 expression on inflammatory cells was associated with a favorable prognosis. These authors [30] also reported a better response to ICIs, although the number of studies was limited. Moreover, findings from recent clinical trials suggest that PD-L1 could help selecting patients for ICIs, although the PD-L1 cut-off to be used is not yet defined [35,36]. 

*BRCA1* and *BRCA2* mutations were present in 24.6% (17/69) of tumors in this series, a similar frequency to that reported in The Cancer Genome Atlas Program (TCGA) [15]. We did not observe association between *BRCA1* nor *BRCA2* status with TILs. In contrast, some studies have reported that tumors with *BRCA1* germline mutations had increased iTILs. Thus, Goode et al. [11] reported in their series of mainly advanced-stage HGSOC that the extent of CD8+ TILs differed significantly by mutation status, as 29% of *BRCA1* mutation carriers had high TIL counts (≥20 iCD8+/HPF), compared to only 18% of non-carriers and 15% *BRCA2* mutation carriers. Similarly, Morse et al. [37] found higher counts of CD3+ TILs in tumors with *BRCA1* mutations or with mutations in HRD genes. The series included all histological types and stages but was enriched in advanced-stage HGSOC. Soslow et al. [13] reported an increased number of iTILs (without immunophenotyping) in tumors with *BRCA1* alterations (mutations and promoter hypermethylation; n = 23) than in *BRCA2*+ (n = 8) and *BRCA*- tumors (n = 13). Although this study did not report the stage of the tumors, they were selected from the TCGA series that mainly included advanced stages. The intrinsic heterogeneity of HGSOC may justify our findings. In fact, immune response is an evolutionary process that depends on multiple factors, such as the anatomical site, tumor progression and mutational status [38].

Although some series [39] and meta-analysis [16,40] have reported an association of *BRCA1/2* mutation and good prognosis, we did not observe this association. However, as previously stated, most studies included advanced-stage HGSOC. In the meta-analysis reported by Bolton et al. [14], the authors observed a better prognosis at 5 years of *BRCA1/2* mutated tumors, but the association was lost when the analysis was restricted to stage I and II OC, even though the series included all histological types. In addition, it has been suggested that the survival advantage associated with *BRCA* status decreases over time [40,41].

In this series, we observed low TMB among early-stage HGSC. Thus, only 15% of tumors had >10 mut/Mb, but three of them had less than 11 mut/Mb. Several studies have analyzed TMB mostly in advanced HGSC, using different methodologies, but no study has focused exclusively on early stages. Some authors have re-evaluated data from the TCGA and have observed a median TMB of around 2 mut/Mb. Zhong et al. [18] reported 5.2 mutations/Mb (range 0–39 mutations/Mb) in 88 HGSC (15% in early stage), but only four (4.5%) had more than 10 mutations/Mb. The authors did not study the differences among stages. Liu et al. [17] analyzed 45 recurrent advanced HGSC and observed a median TMB of 3.4 mut/Mb. The authors did not observe any case with TMB > 10 mut/Mb. Zhang et al. [20] studied 65 ovarian cancer patients (50 HGSC, 20% stage I and II) and found a median TMB of 4.1 mut/Mb (0.6–23.2). The authors did not analyze differences among histological types or stages.

In our series, TMB was not associated with prognosis after using two cut-offs, the median TMB and >10 mut/Mb. Studies analyzing TCGA data have observed that tumors with a higher TMB, as defined by the median value of about 2 mut/Mb, had a better prognosis [42]. Yang et al. [19] reported that stage III HGSOC from 20 long-term survivors had an increased somatic mutation burden when compared with 21 short-term survivors, but TMB was low in both groups (median 1.62 vs. 1.22 nonsynonymous mutations/Mb). 

Regarding therapy response, Liu et al. [17] did not observe an association between TMB and survival or response to ICIs in their series of recurrent heavily treated tumors. Cristescu et al. [43] re-analyzed TMB by WES in 12 clinical trials of advanced HGSOC and, using a cut-off of 175 mut/exome, observed high TMB in 12/293 (4.1%) tumors (histological type not specified). Responses to pembrolizumab were observed in 2 of 12 (16.7%) tumors with high TMB and in 21 out of 281 (7.5%) tumors with low TMB. 

TMB in our series did not differ between HGSOC with and without *BRCA* mutations. In contrast, Liu et al. [17] reported that TMB was significantly higher in *BRCA1/2* mutated advanced HGSC (n = 11, mean around 6 mut/MB) than in wild-type tumors (n = 53, mean around 3 mut/MB), but the analysis included all histologies. 

One limitation of this study was the use of TMA for semiquantitative TIL estimation. Future studies using whole slide images and digital quantitation would help in stablishing a practical robust threshold for prognostication. An additional limitation was that not all tumors in this series were molecularly characterized due to poor DNA quality associated with old samples. 

On the other hand, major strengths in this study included a relatively large series of tumors in an early stage and centralized histotyping using both morphological and immunohistochemical features. 

The assignation of the histological type is not difficult in most ovarian carcinomas. However, there are tumors with an ambiguous morphology, mainly high-grade tumors, where histotyping errors have been reported in up to 30% of cases [44]. For this reason, the use of immunohistochemistry is recommended to refine histological typing. Thus, the use of a short immunohistochemical panel including WT1, p53, Napsin A and progesterone receptor, together with the morphological evaluation, provides an excellent agreement among pathologists [21]. 

## 4. Materials and Methods

### 4.1. Pathology and Immunohistochemistry

This series of HGSC comes from the RECLAMO study (acronym derived from the project titled Molecular Reclassification of Early Stages of Ovarian Cancer: predictive and prognostic impact) from the Spanish Group of Ovarian Cancer Research (GEICO). Cases had paraffin-embedded tumor samples available and >5 years of clinical follow-up. Approval for the RECLAMO study was obtained from the Local Ethics Committees and written informed consent was obtained from all study participants [2].

Immunohistochemistry (IHC) of tissue microarrays (TMAs) was performed as previously reported [2,45,46]. TMA cores of 1 mm diameter were evaluated for the presence of CD4+ (clone 4B12, Agilent, Santa Clara, CA, USA) and CD8+ (clone C8/114B, Agilent, Santa Clara, CA, USA) lymphocytes. Since two cores were evaluated in most tumors, the average count per core was used for statistical analysis. 

PD-L1 IHC testing was performed using the DAKO 22C3 PharmDx (Agilent, Santa Clara, USA) assay according to the manufacturer’s instructions. PD-L1 IHC slides were interpreted using the tumor proportion score (TPS), which is the number of PD-L1 stained tumor cells divided by the total number of viable tumor cells, multiplied by 100. A TPS ≥ 1 was considered positive for PD-L1.

### 4.2. BRCA1 and BRCA2 Mutation Analysis

*BRCA1* and *BRCA2* gene status was evaluated using the Oncomine BRCA Research Assay Panel (Ion Torrent, Thermo Fisher Scientific, Waltham, MA, USA). This assay is based on highly multiplexed amplification that allows the detection of *BRCA1* and *BRCA2* gene variants in somatic and germline samples. The Ion Chef Instrument (Ion Torrent, Thermo Fisher Scientific, MA, USA) was used for the automated library preparation. Next-generation sequencing (NGS) libraries were sequenced with the Ion S5 using the Ion 530 Chef Kit (Thermo Fisher Scientific, Waltham, MA, USA). DNA sequencing data were accessed through the Torrent Suite program (5.12 version—Thermo Fisher Scientific, Waltham, MA, USA). Reads were aligned with the GRCh37-hg19 human reference genome, and potential mutations and copy number alteration were called using the Ion Reporter™ Cloud Software (5.16 version—Thermo Fisher Scientific, Waltham, MA, USA). These processes were performed following the manufacturers’ instructions. In addition, a filter analysis was incorporated selecting only those variants with >500 reads and an allelic frequency >5%. The variant calls were manually checked using the integrative genomics viewer (IGV; Broad Institute, Cambridge, MA, USA); germline and somatic pathogenic or probably pathogenic mutations were not differentiated since normal tissue was not available for most tumors.

### 4.3. TMB

For DNA extraction, paraffin blocks were punched within the tumor area selected in hematoxylin and eosin stained tissue sections to ensure a minimum of 30% of tumor cells. DNA was extracted using QIAamp DNA FFPE Tissue Kit (Qiagen, Valencia, CA, USA) following the manufacturer’s instructions. The Qubit dsDNA Broad-Range assay kit (Invitrogen, Waltham, MA, USA) was used to quantify DNA and qualitative assessment was performed with the Tape Station 2200 and Genomic DNA Kit (Agilent, Santa Clara, CA, USA).

The Oncomine Tumor Mutation Load Assay Panel (Ion Torrent, Thermo Fisher Scientific, Waltham, MA, USA) was used to assess TMB as previously reported (22). The assay covers 1.65 Mb across 409 relevant oncogenes across major cancer types. The Ion Chef Instrument (Ion Torrent, Thermo Fisher Scientific, Waltham, MA, USA) was used for automated library preparations. NGS libraries were sequenced in an Ion S5 using the Ion 540 Chef Kit (Thermo Fisher Scientific, Waltham, MA, USA). DNA sequencing data were accessed through the Torrent Suite program (5.12 version—Thermo Fisher Scientific, Waltham, MA, USA). Reads were aligned with the GRCh37-hg19 human reference genome, and alterations were called using the Ion Reporter™ Cloud Software (5.16 version—Thermo Fisher Scientific, Waltham, MA, USA), according to the manufacturer’s instructions. This platform uses germline filtering with 1000 genomes, 5000 exomes and ExAC databases; nonsynonymous variants are accurately called and assessed for TMB (mutations/Mb). In addition, an analysis filter that increased the value of the minimum allele frequency from 0.05 to 0.1 was incorporated because the high degradation of old paraffin samples produces a large number of low-frequency variants that can distort the results.

### 4.4. Statistical Analysis

Differences in continuous variables were assessed using the Wilcoxon rank-sum test and differences between categorical variables were assessed using the Fisher test for contingency tables.

Relapse-free survival was calculated as the time interval between the diagnosis of HGSOC and first confirmed sign of disease relapse. Overall survival was defined as the time interval between the diagnosis of HGSOC and the date of death or end of follow-up. For survival analysis, the Kaplan–Meier method was used to estimate relapse-free survival and overall survival curves. Only *p* values < 0.05 were considered significant. Finally, multivariate Cox proportional hazard models were carried out to assess hazard ratios associated with each significant risk factor.

## 5. Conclusions

In summary, our results suggested a prognostic role of iTILs in early-stage HGSOC. The role of PD-L1, BRCA status and TMB remains to be established, since most studies were not focused on these stages, included patients with different clinico-pathological characteristics and used different methodologies and cut-offs. 

## Figures and Tables

**Table 1:** Features of our series.

			Percentage
Age at diagnosis			
n = 124	median	56	
	mean	57	
	IQR	49–64	
FIGO stage			
n = 124	IA, IB	27	21.8
	IC	59	47.6
	IIA, IIB	38	30.6
PD-L1			
n = 123	Negative	113	91.9
	Positive	10	8.1
*BRCA1*			
n = 69	Wild type	58	84.1
	Mutated	11	15.9
*BRCA2*			
n = 69	Wild type	63	91.3
	Mutated	6	8.7
TMB			
n = 40	median	5.04	
	mean	6.06	
	IQR	3.4–6–7	
Relapsed			
n = 123	Yes	44	35.8
	No	79	64.2
Deceased			
n = 123	Yes	27	22
	No	96	78

**Table 2:** Summary statistics of TILs and proportion of TILs in each category for the whole series. Tumors with no (negative), moderate (1–20) and high (>20) TILs. iTIL, intraepithelial tumor-infiltrating lymphocytes; sTIL, stromal tumor-infiltrating lymphocytes.

	Median	Mean	IQR	Negative	1–20	>20
iTIL CD4+	5	5.95	0–2	85 (70.2%)	23 (19%)	13 (10.7%)
sTIL CD4+	19.9	18	0–25.5	41 (33.9%)	50 (41.3%)	30 (24.8%)
iTIL CD8+	26	49.6	6.9–65.3	15 (12.5%)	32 (26.7%)	73 (60.8%)
sTIL CD8+	16.3	31.4	7.5–40.9	10 (8.3%)	54 (45%)	56 (46.7%)

## Data Availability

The datasets used and/or analyzed during the current study are available from the corresponding author upon reasonable request.

## Supplementary Materials

The following supporting information can be downloaded at: https://www.mdpi.com/article/10.3390/ijms241311183/s1.
