# Supplementary material for: The Prognostic Significance of Tumor-Infiltrating Lymphocytes, PD-L1, BRCA Mutation Status and Tumor Mutational Burden in Early-Stage High-Grade Serous Ovarian Carcinoma—A Study by the Spanish Group for Ovarian Cancer Research (GEICO)
Source: Int J Mol Sci. 2023 Jul 6;24(13):11183. doi: 10.3390/ijms241311183 (PMC10342764; doi:10.3390/ijms241311183)
Supplement: Supplementary file 1 [file ijms-24-11183-s001.zip › Supplementary Table S1.pdf]

Surgical staging procedures performed and adjuvant treatment			
	**YES**	**NO**	**UNKNOWN**
Omentectomy	101(80,8%)	18(14,4%)	6(4,8%)
Biopsy or resection of any adhesions close to the tumor	78(62,4%)	24(19,2%)	23(18,4%)
Blind biopsy of the bladder peritoneum and pouch of Douglas	53(42,4%)	54(43,2%)	18(14,4%)
Blind biopsy of the left parietocolic space	47(37,6%)	54(43,2%)	24(19,2%)
Blind biopsy or brush biopsy of the right diaphragmatic dome	37(29,6%)	65(52%)	23(18,4%)
Blind biopsy of the wall of the pelvic peritoneum and the local	45(36%)	56(44,8%)	24(19,2%)
Aortic lymphadenectomy	6(4,8%)	4(3,2%)	115(92%)
Was relaparotomy performed?	11(8,8%)	113(90,4%)	1(0,8%)
Presence of ascites	1(0,8%)	10(8%)	114(91,2%)
Gross residual tumor	9(7,2%)	101(80,8%)	15(12%)
Incidental finding in prophylactic salpingo-oophorectomy	0	9(7,2%)	116(92,8%)
Broken capsule before surgery	0	0	125
Adjuvant treatment	115(92%)	7(5,6%)	3(2,4%)

Number of chemotherapy cycles	N
1	1(0,8%)
2	1(0,8%)
3	3(2,4%)
4	28(22,4%)
5	1(0,8%)
6	62(49,6%)
8	1(0,8%)
Unknown	28(22,4%)

Type of chemotherapy	N
Carboplatin + paclitaxel	106(84,8%)
Carboplatin monotherapy	6(4,8%)
Clinical trial Carboplatin + Paclitaxel +/-BIBF 1120	1(0,8%)
Carboplatin + Cyclophosphamide	1(0,8%)
Clinical trial Carboplatin + weekly taxol + Bevacizumab	1(0,8%)
Unknown	10(8%)
