# Supplementary figures and images for: The Prognostic Significance of Tumor-Infiltrating Lymphocytes, PD-L1, BRCA Mutation Status and Tumor Mutational Burden in Early-Stage High-Grade Serous Ovarian Carcinoma—A Study by the Spanish Group for Ovarian Cancer Research (GEICO)
Source: Int J Mol Sci. 2023 Jul 6;24(13):11183. doi: 10.3390/ijms241311183 (PMC10342764; doi:10.3390/ijms241311183)

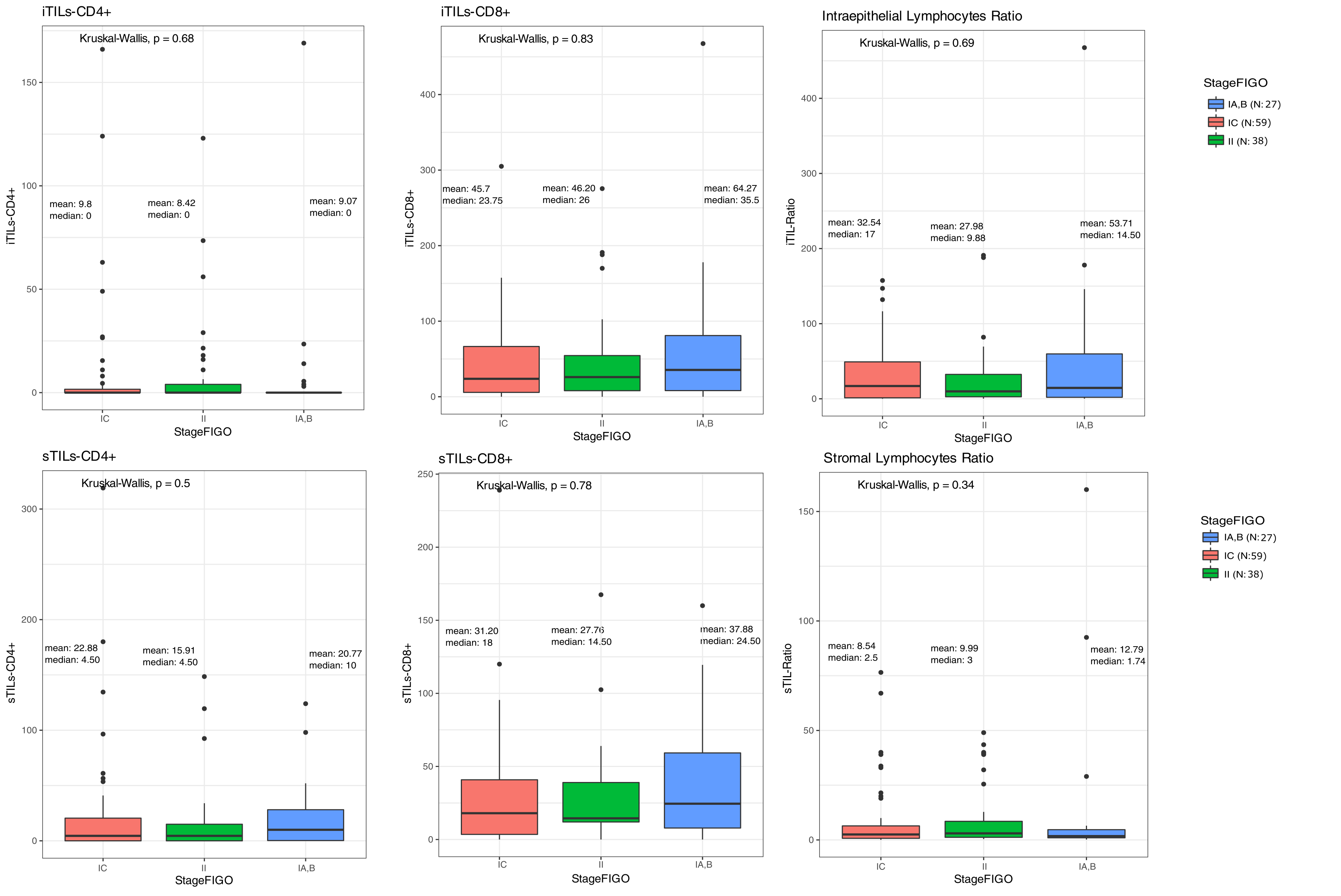

Supplement: Supplementary file 1 [file ijms-24-11183-s001.zip › supplementary figure S1.png]

TMB Level    HIGH    LOW

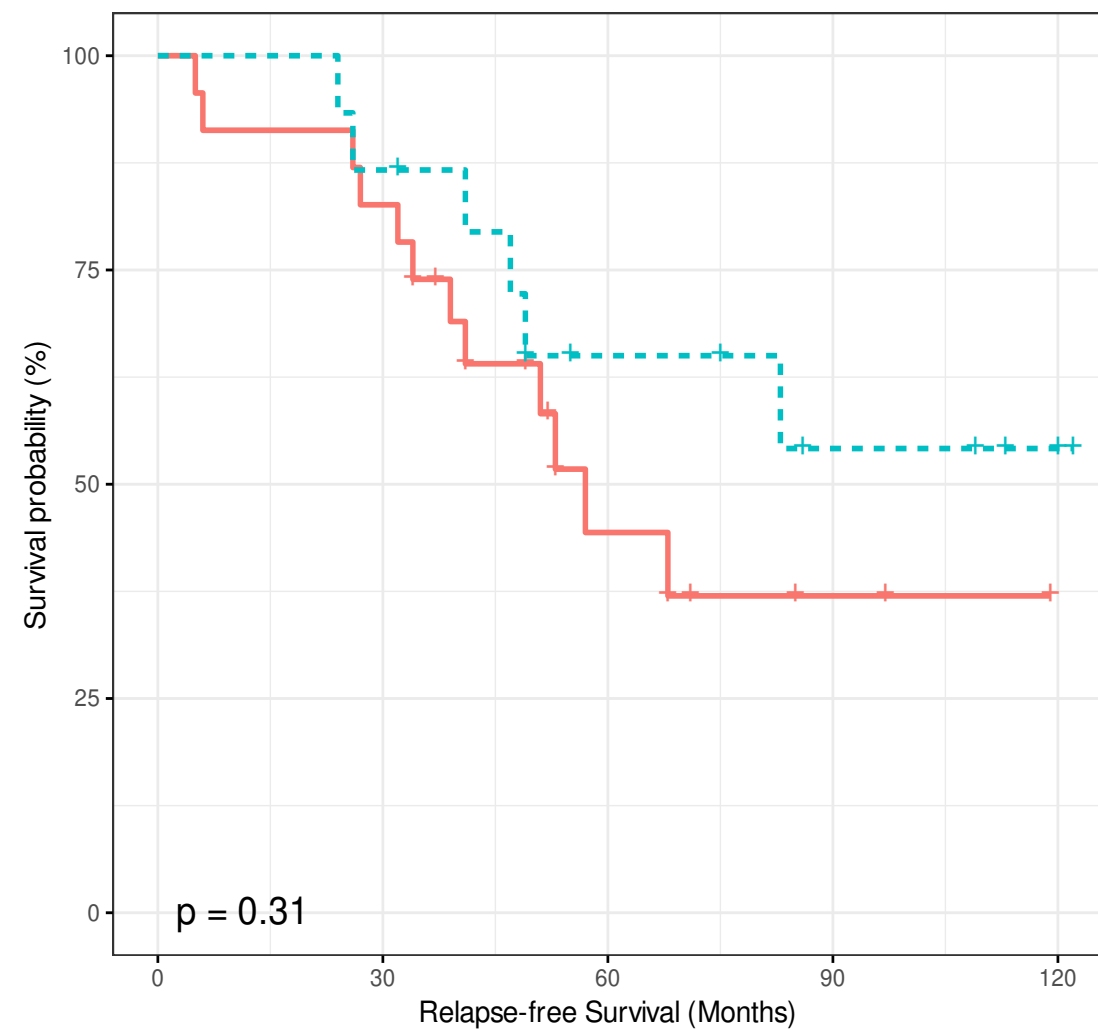

TMB Level    HIGH    LOW

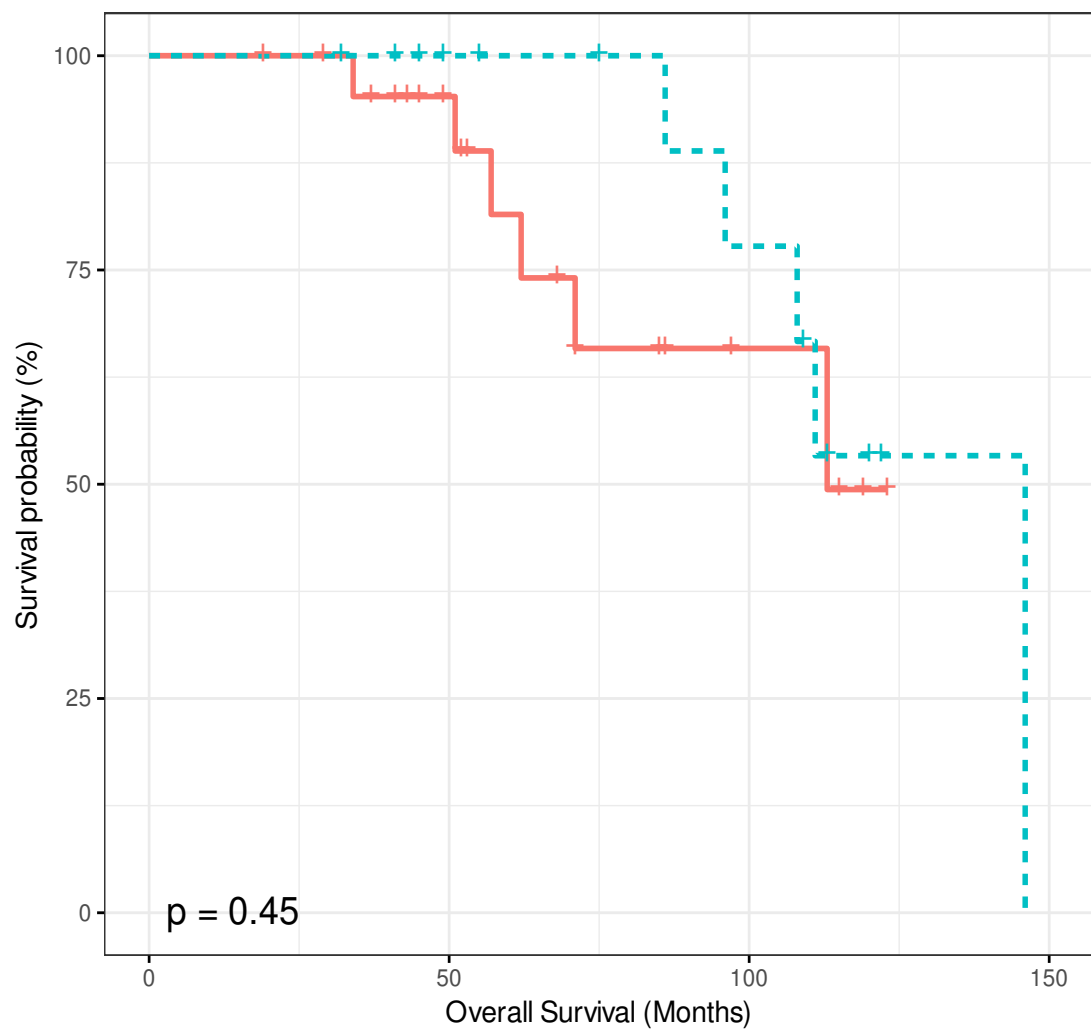

Supplement: Supplementary file 1 [file ijms-24-11183-s001.zip › Supplementary Figure S10_KM_TMB.pdf]

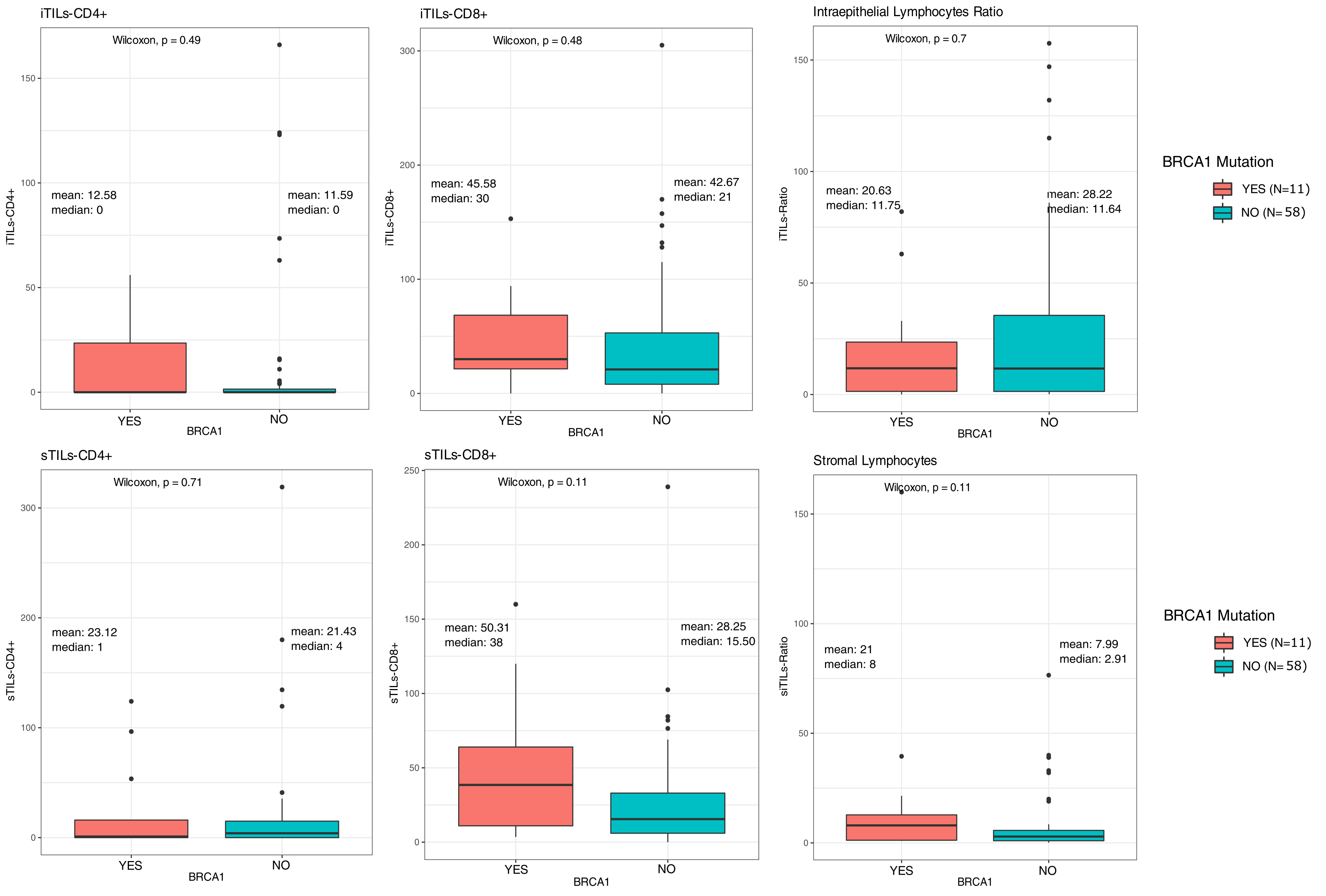

Supplement: Supplementary file 1 [file ijms-24-11183-s001.zip › supplementary figure S2.png]

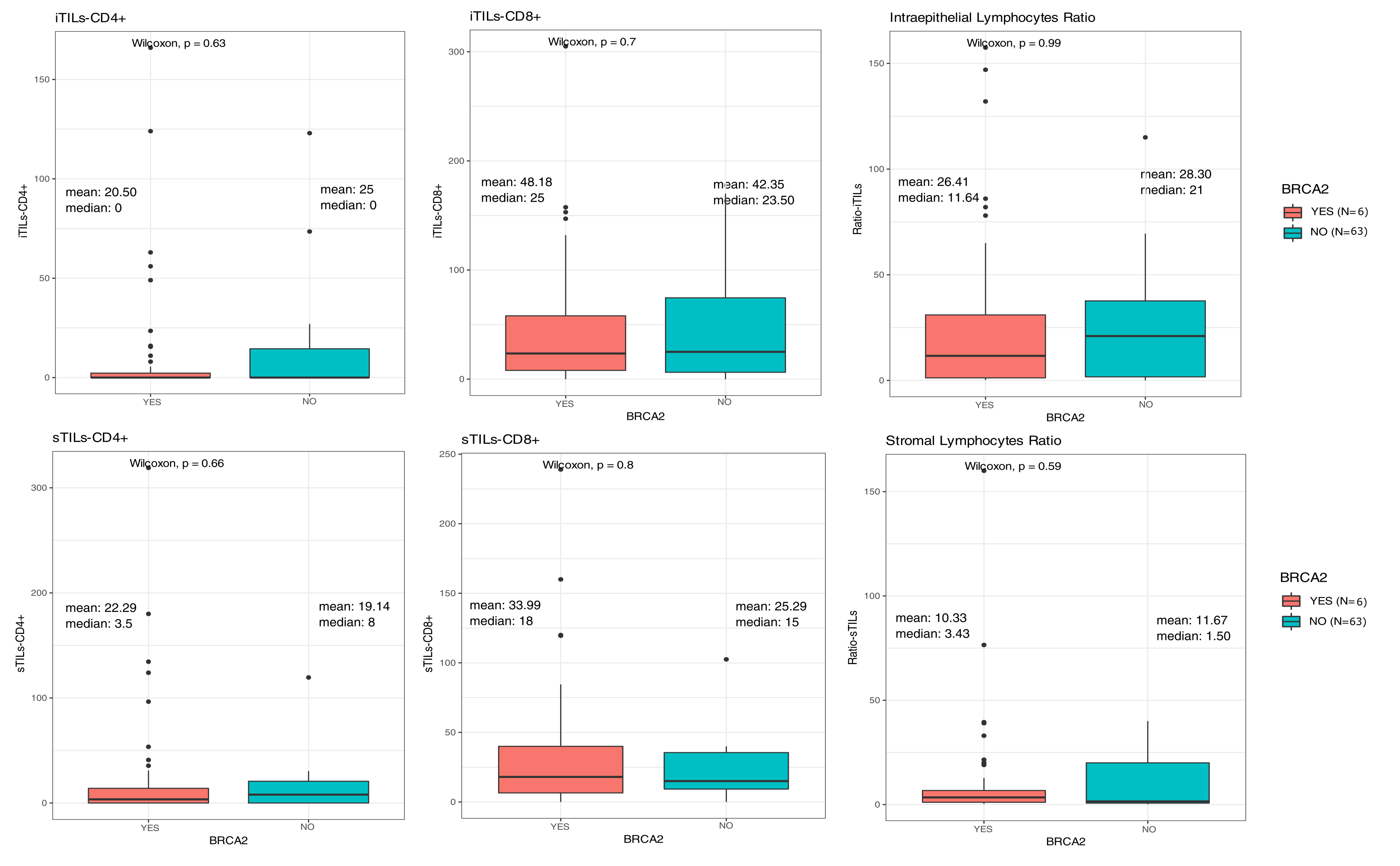

Supplement: Supplementary file 1 [file ijms-24-11183-s001.zip › supplementary figure S3.png]

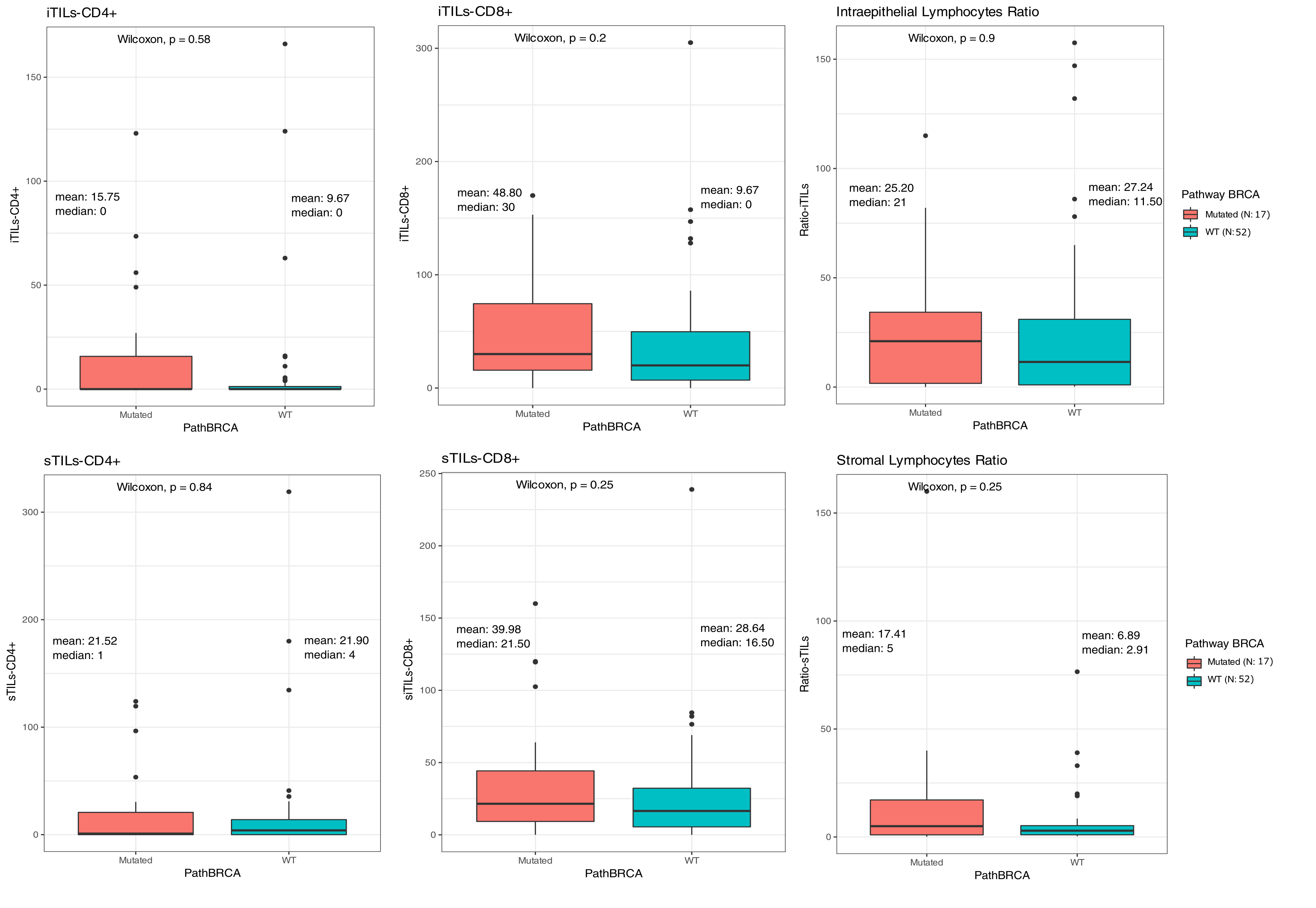

Supplement: Supplementary file 1 [file ijms-24-11183-s001.zip › supplementary figure S4.png]

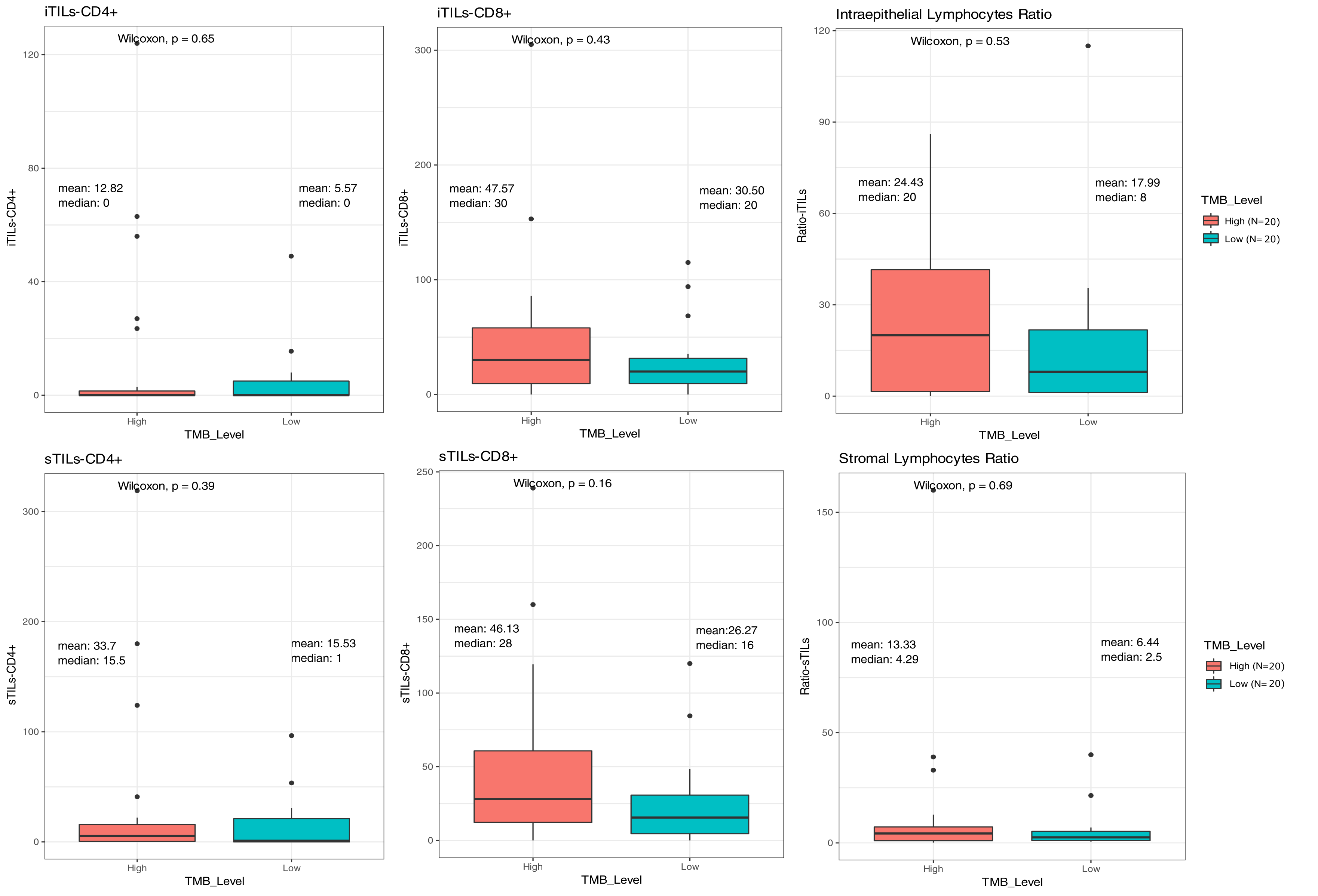

Supplement: Supplementary file 1 [file ijms-24-11183-s001.zip › supplementary figure S5.png]

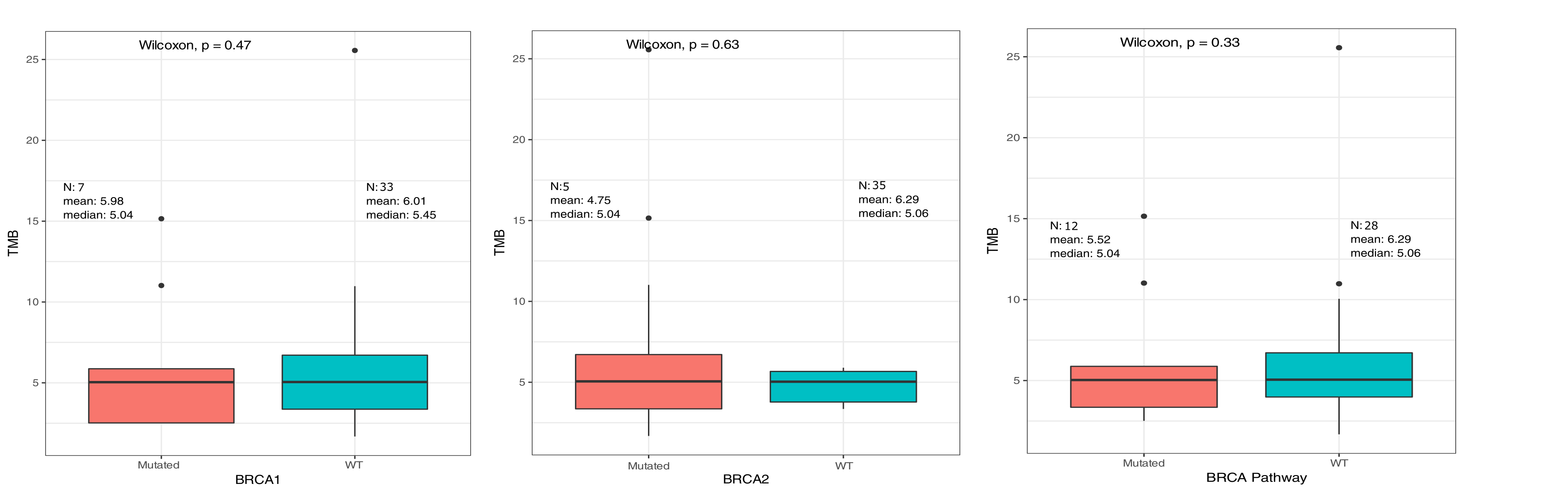

Supplement: Supplementary file 1 [file ijms-24-11183-s001.zip › supplementary figure S6.png]

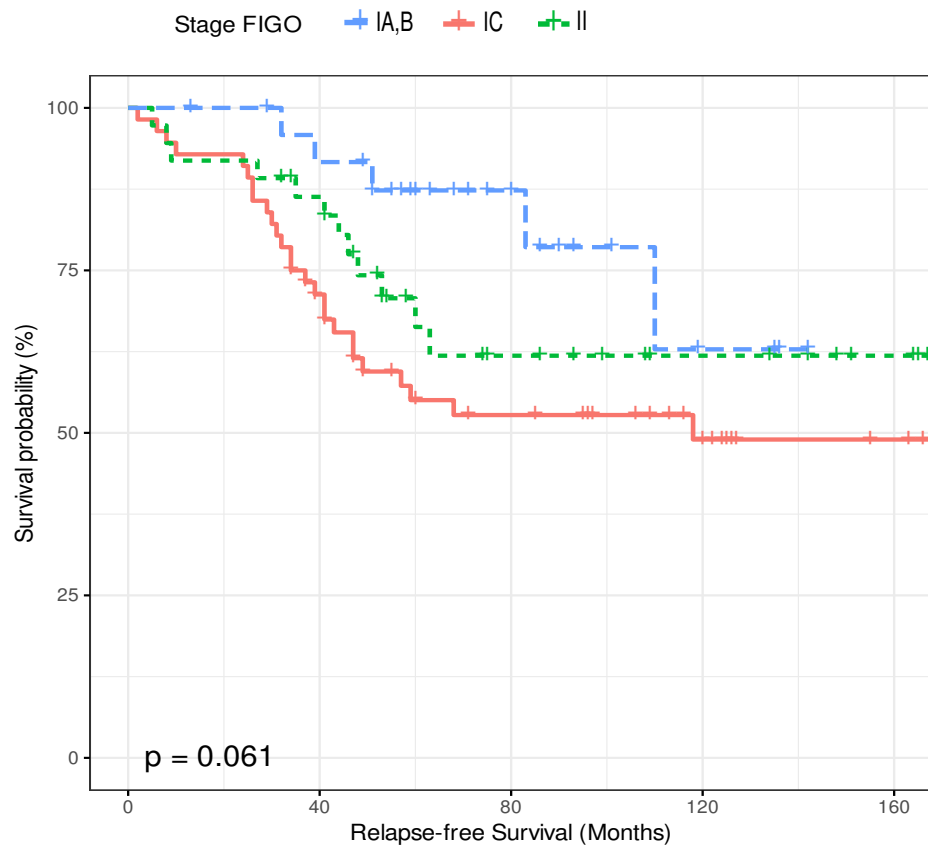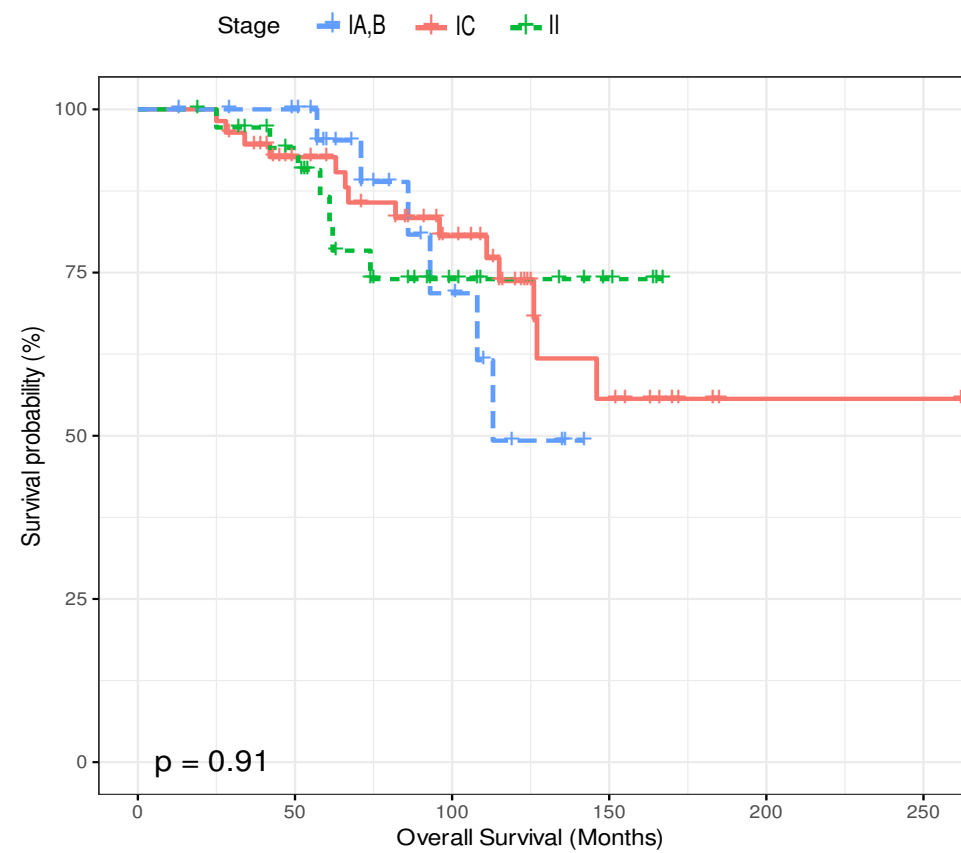

Supplement: Supplementary file 1 [file ijms-24-11183-s001.zip › Supplementary Figure S7_KM_Stage.pdf]

PD-L1 Expression    NO    YES

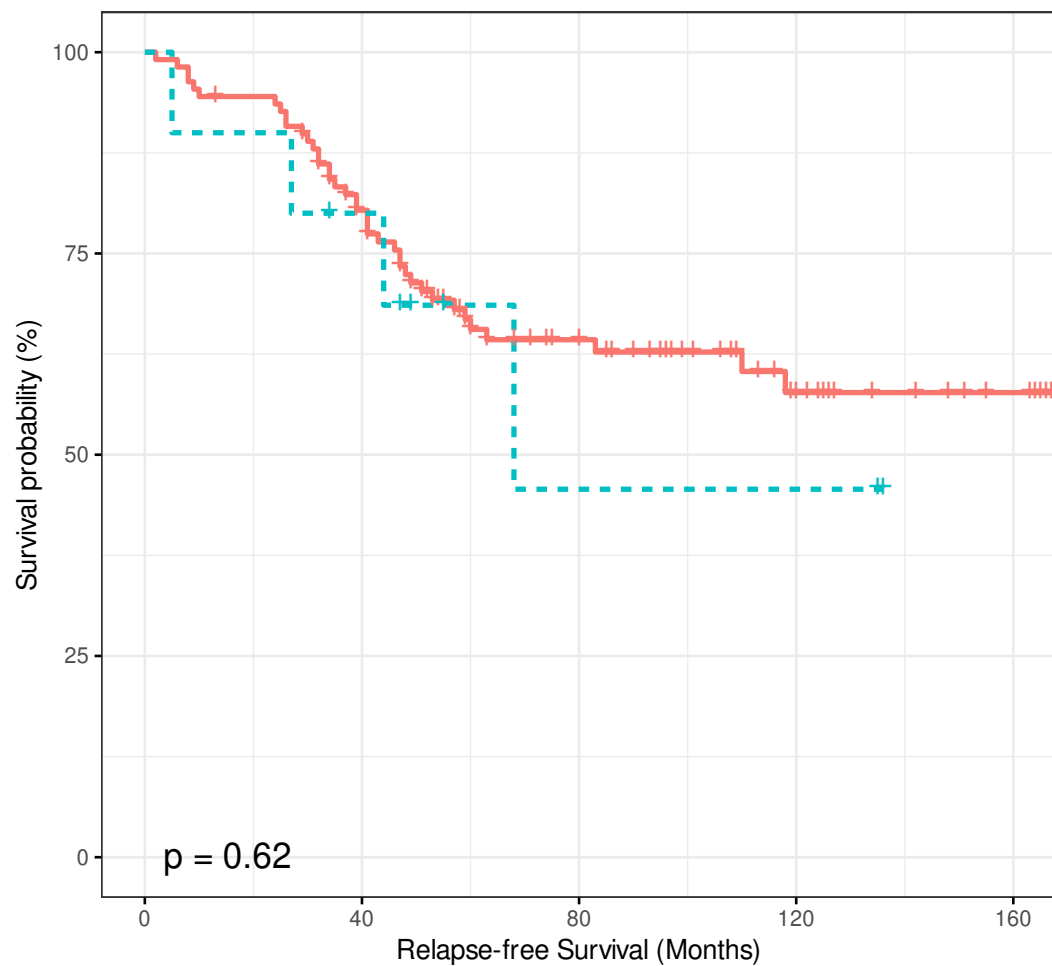

PD-L1 Expression    NO    YES

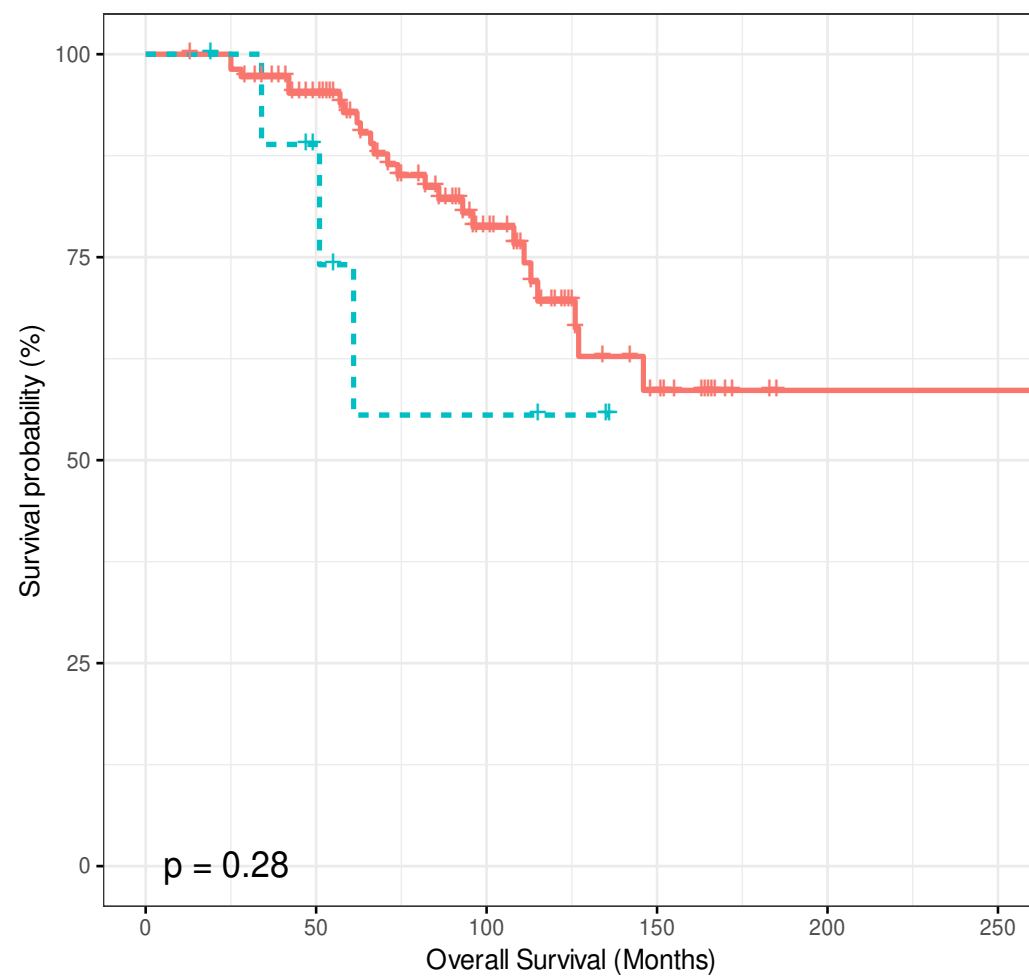

Supplement: Supplementary file 1 [file ijms-24-11183-s001.zip › Supplementary Figure S8_KM_PDL1.pdf]

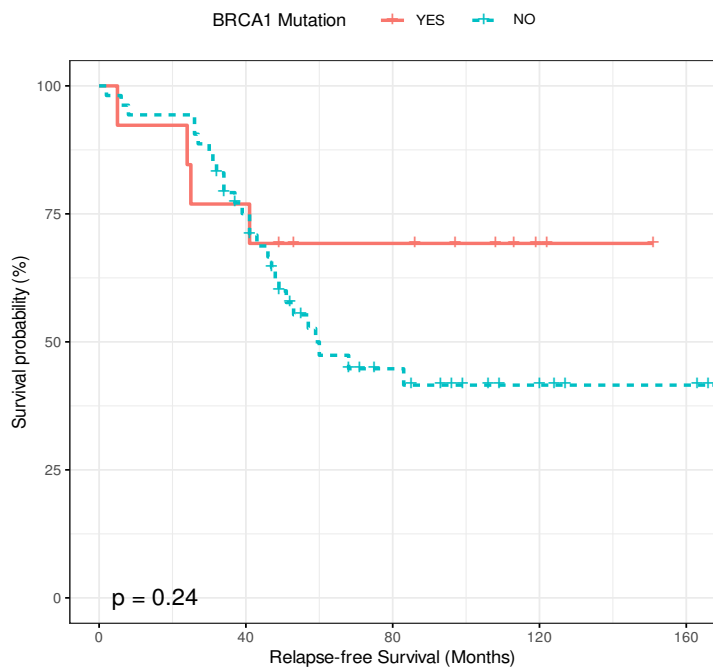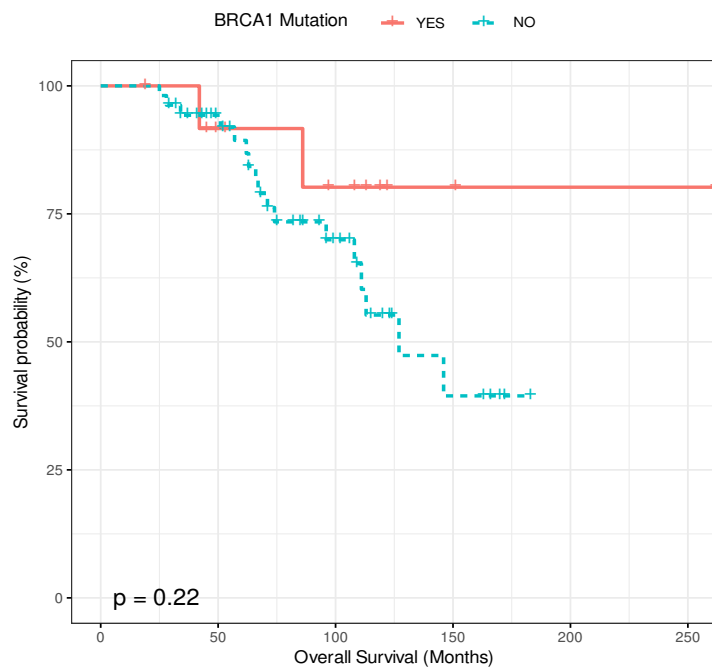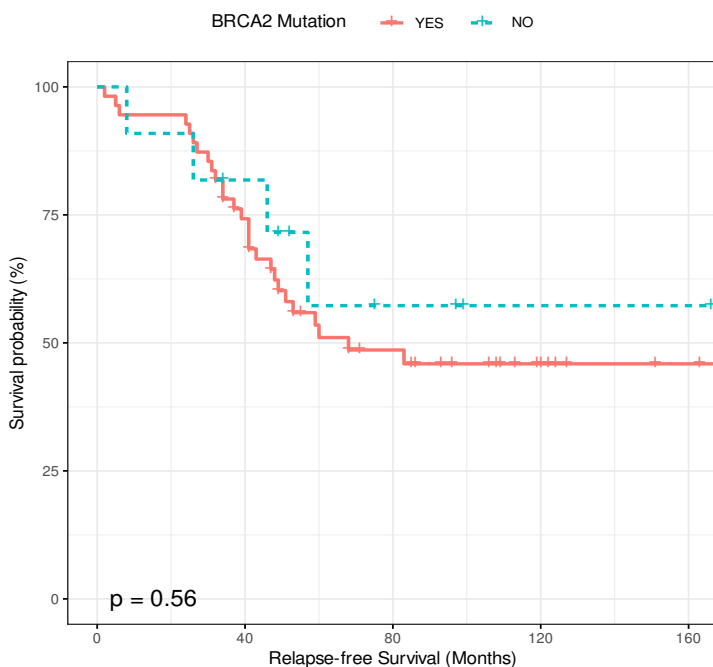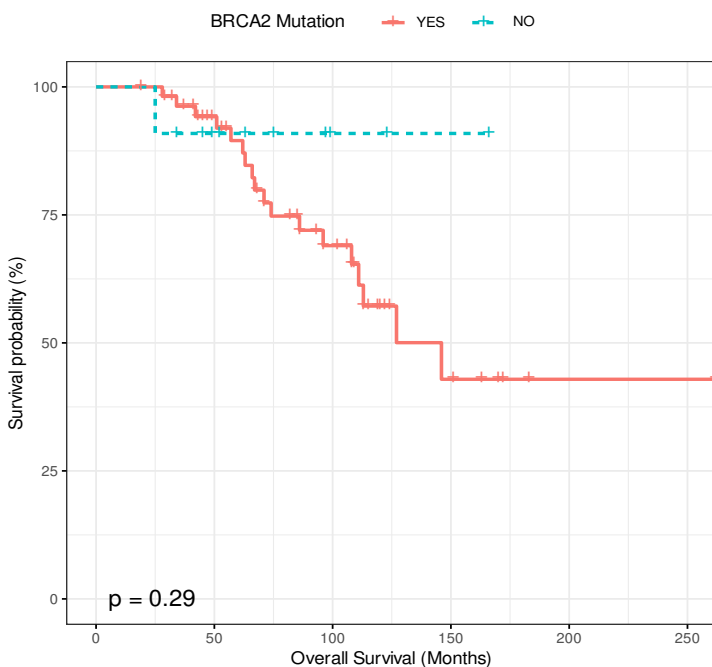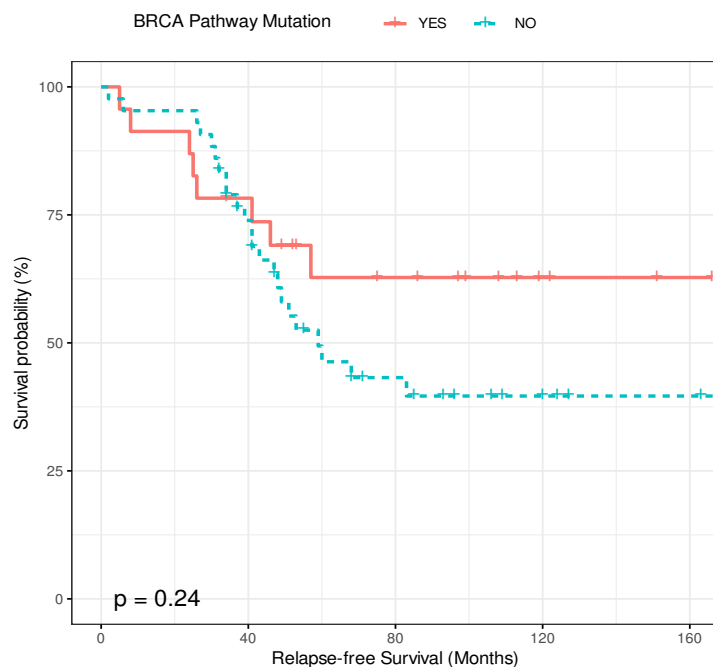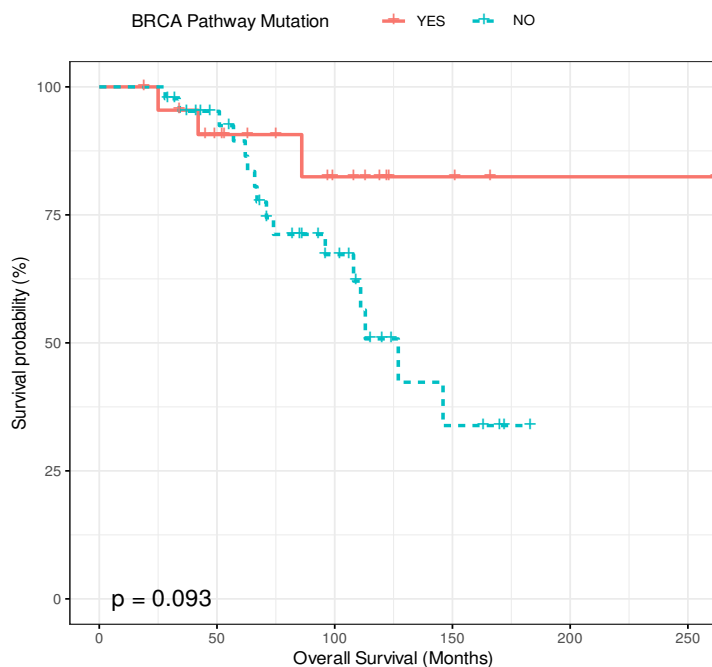

Supplement: Supplementary file 1 [file ijms-24-11183-s001.zip › Supplementary Figure S9_KM_BRCA_Conjunto.pdf]
